# Effect of Coronavirus Disease 2019 on the Psychology and Behavior of Patients on Methadone Maintenance Treatment in Wuhan, China: A Clinical Observational Study

**DOI:** 10.3389/fpsyt.2021.653662

**Published:** 2021-03-30

**Authors:** Xuebing Liu, Xiaohan Jin, Yingying Zhang, Lin Zhang, Yi Li, Jun Ma

**Affiliations:** ^1^Research Center for Psychological and Health Sciences, China University of Geosciences, Wuhan, China; ^2^Affiliated Wuhan Mental Health Center, Tongji Medical College of Huazhong University of Science and Technology, Wuhan, China; ^3^School of Nursing, Xinyang Vocation and Technical College, Xinyang, China

**Keywords:** COVID-19, MMT, psychology, behavior, Wuhan

## Abstract

Methadone maintenance treatment (MMT) is a national strategy adopted for the treatment of heroin dependency in China. The sudden outbreak of coronavirus disease 2019 (COVID-19) and the resultant social isolation in Wuhan have also had a significant negative impact on local patients undertaking MMT. In this study, 76 patients on MMT from the Wuhan First Health Clinic were selected as the research objects to investigate the effect of COVID-19 on the levels of stress, anxiety, and depression, as well as the withdrawal symptoms and craving and substance use. Our results revealed that, during the outbreak, the Perceived Stress Scale (PSS) score, HAMA score, HAMD score, and craving in the included patients was significantly higher than that at the baseline and post-epidemic (*p* < 0.01, *p* < 0.01, *p* < 0.01, *p* < 0.01, respectively); the positive rate of morphine and methamphetamine urine test was significantly lower than that the post-epidemic (*p* = 0.01, *p* < 0.01, respectively); the amount of tobacco used during the outbreak and the post-epidemic period was significantly higher than that at the baseline (*p* < 0.01, *p* < 0.01, respectively), while the amount of alcohol consumed at the outbreak was significantly higher than at the baseline and the post-epidemic (*p* < 0.01, *p* < 0.01, respectively). The negative emotions aroused by the COVID-19 outbreak and the accompanying social isolation to patients on MMT are an important factor of negative reinforcement that adversely affected the patients' craving for drugs and the consumption of legal substances. This finding suggests the need to strengthen the psychological counseling for patients on MMT during severe epidemic, actively alleviating the negative emotions, reducing the risk of substance abuse, and strengthening drug management after the epidemic to prevent the rise of substance (legal or illegal) abuse.

## Introduction

Heroin dependence is a disease characterized by repeated heroin use, compulsory drug-seeking behavior, increased tolerance, and withdrawal symptoms after stopping the use of the drug (1). Methadone maintenance therapy (MMT) is an extremely effective treatment approach for heroin dependency (2), which has been adopted as the national strategy for combating heroin dependency among the Chinese communities (3, 4). Under the guidance of this strategy, a certain amount of methadone was administered to the heroin-addicted patients for free in order to reduce the harm caused by heroin and to help get rid of this habit and make the substance-abuse victims return to the society. Simultaneously, the health status and the employment rate of these patients were improved, and the incidence of acquired immunodeficiency syndrome (AIDS) and hepatitis C reduced significantly (5).

Coronavirus disease 2019 (COVID-19) is a global pandemic and acute respiratory infectious disease caused by a new coronavirus (SARS-CoV-2) (6). The application of methadone maintenance treatment (MMT) is facing severe challenges at the epicenter of the national epidemic in Wuhan, Hubei Province (7). There are presently 17 outpatient clinics set up in Wuhan, with >4,000 people undergoing MMT treatment simultaneously (8). During the COVID-19 epidemic, the local government of the Wuhan City implemented certain measures to control the epidemic, which included restricting the population movement through the traffic control. However, these measures are conflicted by the possible serious consequences of drug overdose and illegal drug trading (7).

Currently, the epidemic is under full control, and it is the post-epidemic era in Wuhan. Recent studies have reported that, during the epidemic, the general population in Wuhan and patients with schizophrenia experienced obvious symptoms such as anxiety, depression, and insomnia (9, 10), with some people also developing post-traumatic stress disorders (11). According to animal experiments and research reports, stress can facilitate addiction, which may lead to increased cravings for substances and increased frequency and degree of relapse behaviors (12). However, the effects of COVID-19 on the behavior of patients undergoing MMT have not been reported adequately. For this reason, in the context of the sudden global outbreak of COVID-19 and the resulting strict social distancing, 76 patients who received MMT from the Wuhan First Health Clinic (which is a part of the Wuhan Mental Health Center that specializes in distributing free methadone to heroin-dependent patients) were treated during the outbreak and during the mid- and post-epidemic periods. To evaluate the withdrawal symptoms, relapse behaviors, and the use of methamphetamine, alcohol, tobacco, and other such substances, we investigated the psychological and behavioral effects of COVID-19 on the MMT-receiving patients to form a basis for clinical intervention.

## Materials and Methods

### Subjects

The 76 subjects recruited in this study were heroin-dependent patients undergoing MMT at the Wuhan First Health Clinic (which is a part of the Wuhan Mental Health Center that specializes in distributing free methadone to heroin-dependent patients). The study inclusion criteria were (1) meeting the Diagnostic and Statistical Manual of Mental Disorder, Fourth Edition (DSM-VI); (2) being registered at the Wuhan First Health Clinic and receiving MMT for >1 year; (3) gender free; (4) age of 20–65 years; (5) adherence to outpatient medicine collection, and the number of outpatient records/month being ≥15 times. The study exclusion criteria included diagnosis of schizophrenia, bipolar disorder, personality disorder, dementia, and other severe cognitive impairment, as well as severe physical diseases and other infectious diseases such as COVID-19.

This study was approved by the ethics committee of Wuhan Mental Health Center. All patients or their guardians provided written informed consent for their participation.

### Study Design and Procedures

On January 25, 2020, Wuhan was locked down in the face of the outbreak of COVID-19; the entire community was asked to remain indoors, traffic control was implemented, and public transportation was suspended throughout the journey, which caused great difficulties to MMT-receiving patients who were under medication. Under such difficult conditions, Wuhan No. 1 Health Clinic insisted on operating normally. Owing to the combined efforts of various community volunteers and anti-drug social workers, MMT was delivered to the patients who had subscribed to MMT in the community but could not visit the outpatient clinics to pick up their medicines. Meanwhile, the basic situation of the patients was investigated every month and their urine drug tests were performed. On April 26, 2020, the COVID-19 patients were cleared, and public transportation resumed in May, after which the public and community controls gradually began to relax. Wuhan has entered the post-epidemic period and has resumed work and production. At this time, patients on MMT can now visit the outpatient clinics to take their medications in person as usual.

The present study was designed as a clinical observation study. In order to evaluate the impact of COVID-19 on the psychology and behavior of included patients, the following tasks were completed during the pre-epidemic period (baseline, October-December 2019), the outbreak period (outbreak, February-April 2020), and the post-epidemic period (post-epidemic, May–June 2020): recording of the urine drug test results to examine the use of drugs (such as heroin and methamphetamine), the number of outpatient visits per month, and the use of tobacco and alcohol. At the same time, the Chinese Opioid Withdrawal Symptom Scale (COWS) and the Visual Craving Scale (VAS) were employed to evaluate the patient's substance withdrawal and drug cravings in order to assist them to investigate about possible relapse. Comparison of the differences in the abovementioned indicators at 3 different time-points, as well as the observations regarding the impact of the epidemic on the psychology and behavior of MMT-receiving patients were also performed.

### Instruments

1. Extraction of the basic data of the patients and the daily drug usage of the methadone patient on the medication management system.
2. The use of a urine drug test board in order to perform urine tests on the patient, followed by evaluation of the use of morphine and methamphetamine in the target patients.
3. A simple self-made questionnaire was used to investigate the patient's use of legal substances (such as alcohol and tobacco), the COWS was used to assess withdrawal symptoms, and VAS to assess the patient's desire for substance use. The Perceived Stress Scale (PSS) was used to assess the severity of participants' psychological stress, the Hamilton Anxiety Scale (HAMA) for the severity of anxiety symptoms, and the Hamilton Depression Scale (HAMD) for the severity of the depressive symptoms.

In order to ensure the consistency of the evaluation results obtained, all psychiatrists participating in the evaluation were provided with uniform training and subjected to consistency evaluation.

### Statistical Analysis

The normally distributed continuous measurement data obtained were expressed as the mean and standard deviation, and the categorical variables as counts. Repeated measures analysis of variance was performed on continuous variables with normal distribution and generalized estimating equations for the rates. The significance level of all statistical tests was set to *p* < 0.05. Data analysis was performed using the IBM SPSS (version 26.0, SPSS Inc., Chicago, IL, USA), and the figures were plotted using the GraphPad Prism software (version 8.4.3; GraphPad Software Inc., La Jolla, CA, USA).

## Results

The general clinical characteristics of the study subjects are shown in Table 1.

**Table 1:** The general clinical characteristics of the MMT-receiving subjects included.

	**Gender** **(female vs. male)**	**Age**	**Years of** **education (years)**	**Drug use** **method^**#**^**	**Course of** **drug addiction** **(years)**	**Years of** **MMT**	**Methadone** **daily dose**	**Monthly follow-up** **times**
Study group (*n* = 76)	26 vs.50	48.53 ± 5.99	9.01 ± 3.01	61 vs. 15	22.32 ± 8.42	14.20 ± 3.28	55.26 ± 13.41	22.22 ± 2.48

# *Intravenous injection vs. inhaling*.

### Differences in the Levels of PSS, HAMA, and HDMA in Patients on MMT in the Three Periods

**Figure 1:**
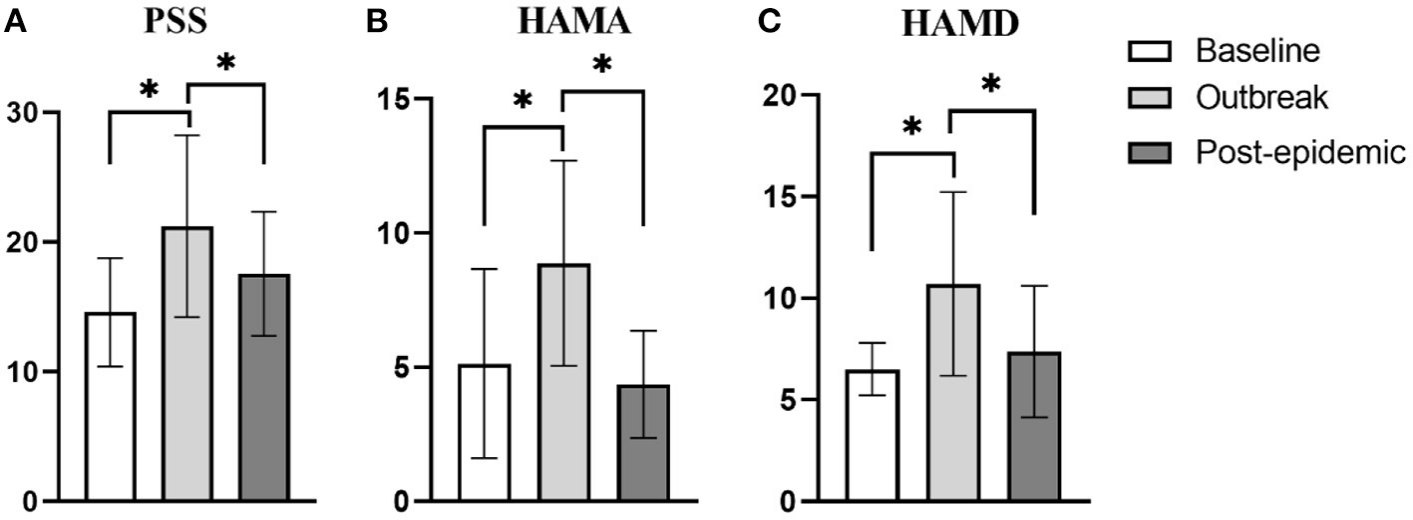
Differences in the score of PSS, HAMA, and HAMD in the three periods. **(A)** The difference in the score of PSS. When the outbreak, the score of PSS was significantly higher than the baseline and post-epidemic (*p* < 0.01, *p* = 0.02, respectively). **(B)** The difference in the score of HAMA. When the outbreak, the score of HAMA was significantly higher than the baseline and post-epidemic (*p* < 0.01, *p* < 0.01, respectively). **(C)** The difference in the score of HAMD. When the outbreak, the score of HAMD was significantly higher than the baseline and post-epidemic (*p* < 0.01, *p* < 0.01, respectively). *p < 0.05.

The influence of time factor on PSS score (Figure 1A), HAMA score (Figure 1B), and HAMD score (Figure 1C) of the included study subjects were statistically significant (*F* = 96.26, *p* < 0.01; *F* = 53.43, *p* < 0.01; *F* = 40.73, *p* < 0.01, respectively).

### Differences in the COWS and VAS in the Three Periods

**Figure 2:**
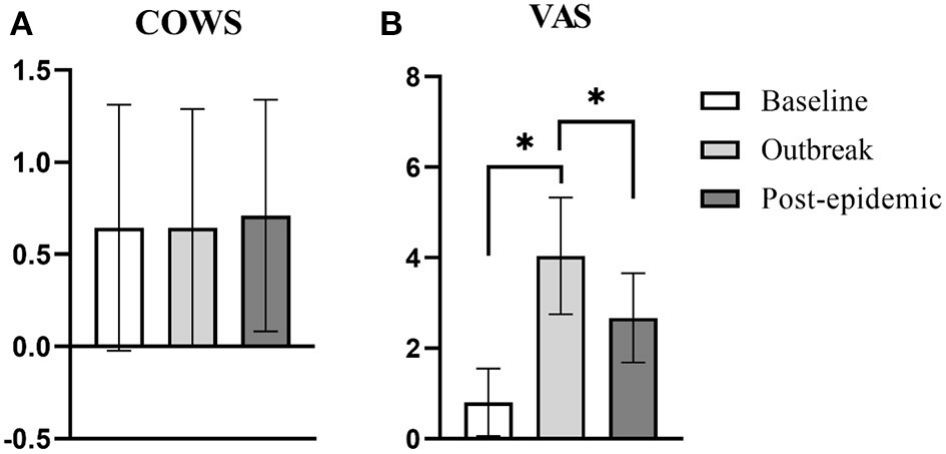
Differences in the score of COWS and VAS in the three periods. **(A)** The difference in the score of COWS in the three periods. There is no difference in the score of COWS in the three periods. **(B)** The difference in the score of VAS in the three periods. When the outbreak, the score of VAS was significantly higher than the baseline and post-epidemic (*p* < 0.01, *p* < 0.01, respectively). *p < 0.05.

The influence of time factor on COWS (Figure 2A) of the included patients were not statistically significant (*F* = 0.53, *p* = 0.534), but VAS (Figure 2B) (*F* = 179.83, *p* < 0.01).

### Difference in the Use of Substances Among the Included Patients in the Three Periods

**Figure 3:**
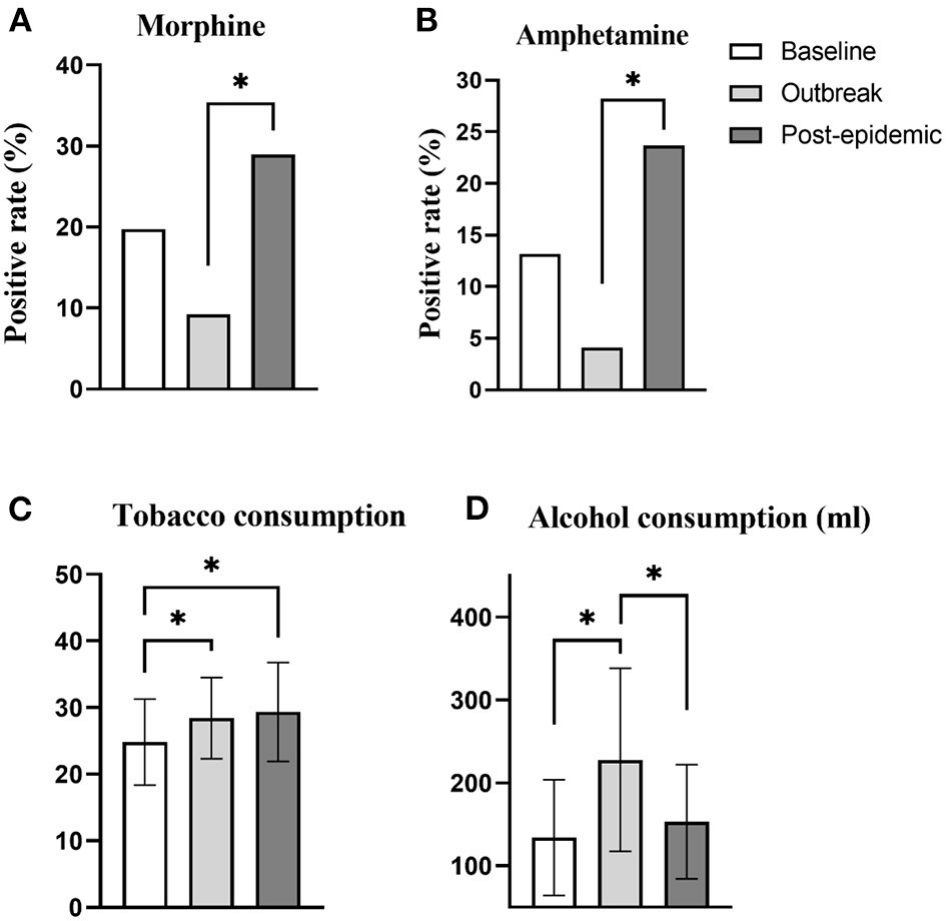
Difference in the use of substances for patients in different time periods. **(A)** Comparison of the positive rate of morphine urine test in different time periods. When the outbreak, the positive rate of morphine urine test was significantly lower than in the post-epidemic (*p* = 0.01). **(B)** Comparison of the positive rate of amphetamine urine test in different time periods. When the outbreak, the positive rate of amphetamine urine test was significantly lower than in the post-epidemic (*p* < 0.01). **(C)** Comparison of differences in tobacco consumption in different periods. During the outbreak and post-epidemic period, the tobacco consumption of the included patients was significantly higher than the baseline (*p* = 0.03, *p* < 0.01, respectively). **(D)** Comparison of differences in alcohol consumption in different periods. When the outbreak, the alcohol consumption of the included patients was significantly higher than the baseline and post-epidemic (*p* < 0.01, *p* < 0.01, respectively). *p < 0.05.

The positive rate of morphine urine test (Figure 3A) and methamphetamine urine test (Figure 3B) had time difference, and the difference was statistically significant (χ^2^ = 2.37, *p* = 0.01; χ^2^ = 3.60, *p* = 0.03, respectively). The influence of time factor on the amount of tobacco (Figure 3C) and alcohol used (Figure 3D) of the included subjects were statistically significant (*F* = 36.67, *p* < 0.01; *F* = 54.45, *p* < 0.01, respectively).

## Discussion

This study ran through 3 periods of the COVID-19 epidemic in Wuhan, China, viz., the before-epidemic stage, the outbreak stage, and the stage of the post-epidemic period. The psychological and behavioral changes of the patients receiving MMT in the community in Wuhan during the epidemic were recorded. For patients in whom the dosage of MMT and the number of outpatient visits were not significantly different, the levels of psychological stress, anxiety, and depression as well as the craving for the use of the original substance or alcohol were significantly higher during the outbreak period than at the baseline and during the post-epidemic periods. The rate of drug relapse during the post-epidemic period was increased significantly; and the amount of tobacco use increased gradually as the epidemic progressed.

### The Impact of COVID-19 on Patients' Relapse Behavior

At 10:00 h on January 23, 2020, in order to curb the further spread of COVID-19, the city of Wuhan was closed, traffic was controlled, and strict social quarantine was implemented. On April 8, 2020, the “Wuhan restart” was announced and the closure was lifted. During the total 76 days of traffic control and social isolation implemented in the lockdown, and >80,000 COVID-19 patients were treated. It is conceivable that the unexpected scope of the COVID-19 outbreak, the speed of transmission, and the severity of the infection, as well as the accompanying strict social distancing all acted as a powerful stress factor for every Wuhan citizen, including the patients on MMT. Our research results also confirm that MMT-receiving patients showed high levels of psychological stress, anxiety, and depression during the outbreak. The adverse effects of stress on substance dependency were mainly mediated through the action of corticotropin-releasing factor and other stress hormones. These hormones weaken the hippocampus and the prefrontal cortex, but strengthen the amygdala, which leads to the development of negative emotions and the desire and lack of executive control that ultimately increases the risk of relapse (13). The results of our study support the general perspective that, during the outbreak, the drug cravings of patients included in this study increased significantly.

Negative reinforcement is one of the two important theories of drug dependency (14). It focuses on emphasizing that, after the drug use, the current bad mood that the addicts are suffering from gets relieved or disappears and that the appearance of negative emotions motivates and maintains the addicts' seeking behavior to continue using the drugs (15). Past studies have shown that, during the COVID-19 outbreak, the incidence and severity of anxiety and depression in the general population generally increases (16, 17). In addition, social isolation is also an important factor that increases crowd anxiety and depression. Studies have reported that severe psychiatric patients in strict social isolation show higher levels of anxiety and depression (10), which is supported by animal studies that have exhibited anxiety and depression-like behaviors in mice under social isolation conditions (18). Equally important, the fear caused by COVID-19, the reduction in income, loneliness, and even the reduction of family members due to the epidemic are all important factors that cause negative emotions in patients. Therefore, whether it is the epidemic or the implementation of social isolation after the epidemic, negative emotions such as anxiety and depression tend to surface. These negative emotions, which act as the main factors of negative reinforcement, increases a drug addict's craving for drugs, as confirmed by our research results.

Nevertheless, the impact of the epidemic on MMT-receiving patients has been negative, which has increased the patient' craving for drugs and the risk of relapse.

### The Impact of COVID-19 on Illegal and Legal Substance Use

In China, the proportion of addicts' smuggling opioids during MMT was recorded at 10.4–28.1% (19, 20), and the proportion of combined abuse of methamphetamine was 12.9–39.6% (19, 21). It is well-known that the consumption of alcohol and tobacco in MMT-receiving patients is extremely common (22–24). Past studies have shown that depression and anxiety can lead to increased alcohol intake (25). Equivalently, the continued presence of stressful events have a positive impact on the increase in the consumption of tobacco (26) and alcohol (27, 28).

Our study discovered that, during the epidemic, the positive rate of urine tests for morphine and methamphetamine was lower (although there was no statistical difference when compared with the baseline value) of the included patients, which indicates that the number of illegal substance users had decreased. This decrease can be explained by the fact that strict social isolation and traffic control led to the blockage of drug circulation channels, which in turn hampered the channels used by the drug addicts to obtain the drugs. With the unblocking of the cities and the restoration of logistics and transportation, the negative emotions and cravings caused by stress have been relieved and satisfied (29) in parallel with the number of users of illegal substances that has also risen.

Different from the changing trend of using illegal substances, as a legal substance, the amounts of tobacco used during the outbreak and the post-epidemic period were significantly greater than that used before the epidemic. Meanwhile, the use of alcohol during the outbreak was significantly greater than that used during the other two periods examined. On one hand, this event benefited from the local government's guarantee of daily consumables and the hard work of the community workers, which enabled the provision of adequate supplies of these materials. On the other hand, as the result of an animal research stated that nicotine contained in tobacco can effectively relieve anxiety and depression caused by chronic stress (30). Clinical studies have also reported that smoking and drinking can relieve anxiety and depression in a patient (31, 32). As mentioned earlier, negative reinforcement means that negative emotions can be relieved after using drugs. During the outbreak of the epidemic, the inclusion of patients with anxiety and depression in a stressful environment that increases their cravings for drugs, but the drugs could not be effectively and adequately supplied. Meanwhile, a more reasonable explanation is that an addict uses sufficient legal substances such as tobacco and alcohol to relieve anxiety, depression, and stress in order to ultimately achieve the purpose of suppressing the cravings.

All addictions or dependence behavior often share similar clinical manifestations, such as the repeated use, compulsory drug-seeking, and loss of control, which involve common psychophysiological mechanisms, including positive reinforcement and negative reinforcement (29). Multi-drug abuse involves a common physiological mechanism: negative emotions form negative reinforcement on almost all substance use, leading to increased use of multiple substances (33). COVID-19 and the accompanying social isolation have induced negative emotions such as stress, depression, and anxiety to strengthen the craving for illegal drugs and increase the consumption of legal substances. This observation suggests the need to strengthen the psychological counseling for patients on MMT during severe epidemic, which actively alleviate the negative emotions, reduce the risk of substance abuse, and strengthen drug management after the epidemic in order to prevent the rise of substance (legal or illegal) abuse.

## Data Availability Statement

The original contributions generated in the study are included in the article/supplementary material, further inquiries can be directed to the corresponding authors.

## Author Contributions

XL and JM made substantial contributions to conception and design of the study. XJ and LZ drafted and revised the manuscript. YZ and JM undertook the data collation, analysis, and picture production. JM and YL gave final approval of the version to be published. All authors contributed to the article and approved the submitted version.

## Conflict of Interest

The authors declare that the research was conducted in the absence of any commercial or financial relationships that could be construed as a potential conflict of interest.
